# *In Vitro* Antibacterial and Phytochemical Screening of *Hypericum perforatum* Extract as Potential Antimicrobial Agents against Multi-Drug-Resistant (MDR) Strains of Clinical Origin

**DOI:** 10.1155/2023/6934398

**Published:** 2023-04-14

**Authors:** Momen M. Sherif, Hussien H. Elshikh, Marwa M. Abdel-Aziz, Mahmoud M. Elaasser, Mohammed Yosri

**Affiliations:** ^1^Department of Microbiology, Faculty of Science, Al-Azhar University, Nasr City, Cairo 11841, Egypt; ^2^The Regional Center for Mycology and Biotechnology, Al-Azhar University, 11787 Nasr City, Cairo, Egypt

## Abstract

**Background:**

The perennial plant *Hypericum perforatum* is widely distributed around the world. It has been used for many years in conventional medicine to treat a variety of illnesses, including stress, mild to moderate depression, and minor injuries. This study examined the antimicrobial activity of the *H. perforatum* total extract and its fractions (n-hexane, ethyl acetate, chloroform, and aqueous) against multi-drug-resistant (MDR) isolates that were gathered from clinical samples, including methicillin-resistant *Staphylococcus aureus* (MRSA), *Enterococcus faecalis*, *Escherichia coli*, and *Klebsiella pneumonia*.

**Materials and Methods:**

Aerial parts of *H. perforatum* were collected and extracted using various solvents and were tested versus different isolated bacterial species. The inhibition zone of tested extracts was detected using an agar diffusion assay, and MICs were measured. Phytochemical analysis of promising *H. perforatum* extract was done using LC-ESI-MS/MS. Ultrastructure examination for the most altered bacteria used transmission electron microscopy. Antioxidant assays were done using DPPH and ABTS scavenging capacity methods. Cytotoxicity was reported versus Vero cells.

**Results:**

Different extracts of *H. perforatum* showed promising antibacterial activity against the pathogens. While the subfractions of the total extract were observed to show lesser inhibition zones and higher MIC values than the total extract of *H. perforatum* against MDR strains, the total extract of *H. perforatum* demonstrated the most potent antimicrobial action with an inhibition zone range of 17.9-27.9 mm. MDR-*K. pneumoniae* was discovered to be the most susceptible strain, which is consistent with the antibacterial inhibitory action of *H. perforatum* whole extract. Additionally, after treatment at the minimum inhibitory concentration (MIC 3.9 *μ*g/ml), the transmission electron microscope showed alterations in the ultrastructure of the *K. pneumoniae* cells. Methanol extract from *H. perforatum* has a CC_50_ value of 976.75 *μ*g/ml.

**Conclusion:**

Future inhibitors that target MDR strains may be revealed by these findings. Additionally, the extracts that were put to the test demonstrated strong antioxidant effects as shown by DPPH or ABTS radical-scavenging assays.

## 1. Introduction

Resistance to antibiotics in pathogenic bacteria is a problem that affects the entire world and is linked to high morbidity and mortality rates. Gram-positive and Gram-negative bacteria have developed multidrug resistance patterns, which have led to infections that are tough to treat or even untreatable with standard medicines [1]. In order to replace existing antimicrobial compounds, it is imperative to investigate novel antimicrobial molecules from all sources; the high cost of producing synthetic drugs and their negative side effects in comparison to naturally derived agents from plants encourage a return to nature [2]. Natural compounds with plant sources are one of the most effective ways to address this issue because of their minimal toxicity, biodegradability, and environmental friendliness when compared to chemical or synthetic agents with antibacterial properties [3, 4]. The many biologically active substances found in plant derivatives encouraged investigators to look at a broader range of potential medicinal applications for their generally safe substances [5].

The most common species of the Hypericum genus and member of the Hypericaceae family, *Hypericum perforatum*, is a rich supplier of flavonoids and is used extensively for therapeutic purposes around the globe [6]. It's possible that the hypericin in *H. perforatum* extract works well against enveloped viruses [7]. Oily Hypericum formulations are applied to manage minor wounds, scars, and skin irritation [8]. Antibacterial and anti-inflammatory properties, as well as enhancement of fibroblast movement and collagen formation, are all factors in how *H. perforatum* treats wounds [9]. *H. perforatum* has a lot of bioactive compounds with anti-inflammatory properties, and more recently, it has primarily been used to treat anxiety and depression in place of traditional antidepressants, with which it shares the inhibition of the uptake of monoamine neurotransmitters [10–13].

Hyperforin and hypericin, which are typically present in the total hydroalcoholic extract of *H. perforatum* in concentrations ranging from 1 to 5% and 0.1 to 0.3%, respectively, are the most significant and characteristic bioactive molecules of this species and are responsible for this plant's pharmacological properties. The whole extract of *H. perforatum* frequently contains other active substances such hyperoside, rutin, quercetin, and various catechins, though their concentrations might vary greatly depending on seasonal variations and the plant's place of origin [14–16]. In the current investigation, seven MDR pathogenic bacteria from clinical samples were tested using an *in vitr*o antimicrobial assay on the entire methanol extract of *H. perforatum* and its fractions.

## 2. Materials and Methods

### 2.1. Plant and Extract Preparation

Aerial parts (flower and leaf) of the *H. perforatum* plant were collected from Belbeis, Elsharkia, Egypt, in March-April 2022, and identified by a botanist in the Faculty of Science at Al-Azhar University using voucher number 946. It was first cleaned with tap water, dried outside, cut into little pieces, and then mechanically pulverized with a blender. After that, it was suspended in methanol for seven days to create the extract. The extracts underwent filtering and evaporation (50°C) before being dried in an oven at 60°C. The entire extract (10 gm of dry powder) was submitted to a bioassay-guided fractionation process that started with water solubilization and progressed via n-hexane, chloroform, and ethyl acetate partitioning in order. Under reduced pressure, each collected fraction was concentrated to produce a black residue [17]. The extraction yield was 31.25, 4.39, 5.71, and 9.83% for methanol, n-hexane, chloroform, and ethyl acetate, respectively.

### 2.2. Isolation and Detection of Harmful Bacteria

Briefly, clinical samples from patients admitted to Al-Zahraa University Hospital and Cairo Specialized Hospital in Cairo city were used to isolate bacterial strains. The samples were cultivated on appropriate agar media, and VITEK 2 was used to identify the bacterial isolates (Biomerieux, New Delhi, India) [18].

### 2.3. Antibiotic Susceptibility Assay

The susceptibility to the commercial antibiotics of the bacterial isolates was evaluated using the disc diffusion method. Antibiotics used against Gram-positive bacteria included cefoxitin, benzyl-penicillin, oxacillin, imipenem, gentamicin, ciprofloxacin, moxifloxacin, inducible clindamycin resistance, erythromycin, clindamycin, vancomycin, tetracycline, fusidic acid, and trimethoprim/sulfamethoxazole. On the other hand, antibiotics used against Gram-negative bacteria included temocillin, ampicillin, amoxicillin/clavulanic acid, ticarcillin, ticarcillin/clavulanic acid, piperacillin, piperacillin/tazobactam, cephalothin, cefuroxime, cefotaxime, ceftazidime, ceftriaxone, cefepime, ertapenem, imipenem, meropenem, amikacin, gentamicin, tobramycin, ciprofloxacin, tigecycline, fosfomycin, nitrofurantoin, pefloxacin, minocycline, colistin, and trimethoprim/sulfamethoxazole (Himedia Labs, Mumbai, India) [19].

### 2.4. Antibacterial Action

To evaluate the extracts' antibacterial effectiveness against microorganisms, an agar well diffusion experiment was employed [20]. Nutrient agar was the culture medium that was used. Wells with a diameter of 6 mm were drilled into the solid agar. The inocula (1.5 × 10^8^ CFU/ml) were dispensed on nutrient agar plates using sterile swabs, and then, 100 *μ*l of extracts was added. The concentration of each of the used extracts was 0.01 g/ml. The plates were then incubated for a further 24 hours at 37°C. After incubation, the zone of growth inhibition for each extract was measured. The MIC of the active ethyl acetate fraction was reported by Khan et al. [21]. The extracts were serially diluted twice. Each inoculum was made in the proper medium for the broth microdilution procedure, its density was adjusted to 0.5 McFarland standards (10^8^ CFU/ml), and its volume was diluted to 1 : 100. On microtiter plates, the MIC was determined following a 24-hour incubation period at 37°C.

### 2.5. Ultrastructural Changes Brought on by *H. perforatum* Whole Extract in *K. pneumoniae* Cells

A concentration of 10^6^*K. pneumoniae* cells was treated with 0.25 micrograms per milliliter of total methanol extract from *H. perforatum*, compared to control cells, and was then left undisturbed for 20 hours to determine their impacts on the ultrastructure. The suspension was rinsed twice with phosphate-buffered saline after being centrifuged down to a pellet. Standard techniques for fixing and embedding biological samples for transmission electron microscopy (TEM) were carried out after these processes, and the JEOL010 instrument was used to analyze the results [22].

### 2.6. Chromatographic Separation of Phytochemicals

Chromatographic separation was carried out on a 5 m C18 column (50 2.0 mm internal diameter; Bohus, Sweden) fitted with an Agilent LC-ESI-MS System (Agilent Technologies, Palo Alto, CA, USA). The temperature of the column was maintained at 40°C. The whole movie lasted for 70 minutes. The mobile phase, which was acetonitrile-water (50 : 50, *v*/*v*) containing 0.1% trifluoroacetic acid, was administered at a flow rate of 0.3 ml/min. When the mass spectrometer was in the positive ion mode, both of its quadrupoles were set to 0.7 full width at half height (FWHM, unit resolution). In addition, the spray voltage was set at 4500 V and the ion tube temperature at 210°C. At 49, 2.0, and 14 arbitrary units, the nitrogen sheath, ion sweep, and auxiliary gases, respectively, were all set. Through infusion experiments, the ESI-MS/MS parameters were altered to create the most deprotonated molecules and the most effective generation of unique fragment ions for all compounds. MS-DIAL V. 3.70 software was used to identify and calculate the relative percentage of molecules [23].

### 2.7. Antioxidant Assay

- For the DPPH radical-scavenging assay, 2,2-di(4-tert-octylphenyl)-1-picrylhydrazyl stable free radicals (DPPH) were added to the test samples in a 96-well plate to cause reaction. The DPPH concentration was held at 300 mM. 30 minutes were spent incubating a reaction volume containing methanol, different extract levels, and DPPH at 37°C. After incubation, the absorbance was recorded using a Tecan microplate reader, USA [24]
- The ABTS scavenging capacity method as a decolorization assay was used to assess the ability of antioxidants to directly react with ABTS radicals according to Ling et al. [25]

### 2.8. Viability Assay for Cytotoxicity Evaluation

In 96-well plates, the Vero cells were planted with 1 × 10^4^ cells and 100 *μ*l of DMEM growth medium. Confluent cell monolayers were placed into 96-well flat-bottomed microtiter plates (Falcon, Jersey, NJ, USA) using a multichannel pipette after being seeded for 24 hours. New DMEM medium containing various amounts of the samples was then added, followed by successive twofold dilutions of the examined specimen. The microtiter plates were incubated for 48 hours at 37°C in a humid incubator with 5% CO_2_. Following crystal violet staining, absorbance was determined at 590 nm [26].

### 2.9. Statistical Analysis

The *t*-test was employed by SPSS software for various analyses of the trials, and all tests were conducted in triplicates.

## 3. Results

### 3.1. Antibiotic Susceptibility

Antibiotic susceptibility tests for two Gram-positive and five Gram-negative bacterial isolates were carried out. MRSA strain was resistant to ten of the nineteen used antibiotics while *E. faecalis* strain was resistant to thirteen of the twenty used antibiotics. Concerning Gram-negative bacterial isolates, for the ratios of the number of resistant antibiotics, the total number of antibiotics was as follows: *E. coli* (18/23), *K. pneumoniae* (19/23), *P. aeruginosa* (16/16), *A. baumannii* (20/23), *Proteus mirabilis* (15/23), and *S. maltophilia* (22/23) (Table 1).

### 3.2. Susceptibility of MDR Isolates to Different *H. perforatum* Extracts and MICs

The n-hexane fraction showed no antibacterial activities against all tested strains except for *P. mirabilis*, while the total extract of *H. perforatum* and ethyl acetate, chloroform, and aqueous fractions revealed variable antibacterial activities against all tested strains except for *A. baumannii*. The antibacterial activities of the total extract of *H. perforatum* and its five solvent fractions were in descending order: total extract > ethyl acetate fraction > chloroform fraction > aqueous fraction > n‐hexane fraction. *P. mirabilis* and *S. maltophilia* were the lowest susceptible pathogens to the aqueous extract with an inhibition zone of 13.4 mm. The total extract of *H. perforatum* showed the maximum diameter of the size zone of inhibition against *K. pneumoniae* (27.9 mm), followed by *E. faecalis* (25.3 mm), MRSA (23.0 mm), *P. aeruginosa* (20.4 mm), *S. maltophilia* (18.3 mm), and *P. mirabilis* (17.9 mm). The n-hexane showed no antibacterial activity except for *Proteus mirabilis* (15.3 mm). Ethyl acetate fraction showed the maximum diameter of the size zone of inhibition against *K. pneumoniae* (24.4 mm), followed by MRSA (22.3 mm), *E. faecalis* (19.8 mm), *P. aeruginosa* and *P. mirabilis* (16.3 mm), and *S. maltophilia* (15.8 mm). The chloroform fractions of *H. perforatum* showed more inhibition zone diameter against *E. faecalis* (20.9 mm), *K. pneumoniae* and *S. maltophilia* (18.6 mm), *P. aeruginosa* (16.35 mm), and MRSA (16.2 mm). Finally, the aqueous fraction showed an inhibition zone against MRSA of 19.3 mm, followed by *E. faecalis* (18.4 mm), *K. pneumoniae* (14.8 mm), and *P. mirabilis* and *S. maltophilia* (13.4 mm). The antibacterial activities are recorded in Table 2. The MIC values of the total fraction were determined as they revealed the maximum level of antibacterial activity. An MIC value of 125 *μ*g/ml of the total fraction was recorded against *P. mirabilis*, 62.5 *μ*g/ml was the MIC value against *S. maltophilia*, 31.25 *μ*g/ml was the MIC value against *P. aeruginosa*, 15.63 *μ*g/ml was the MIC value against MRSA, 3.9 *μ*g/ml was the MIC value against both *E. faecalis* and *K. pneumoniae*, and finally, no MIC value of total fraction was registered against *A. baumannii* as shown in Table 2.

### 3.3. TEM Study

**Figure 1:**
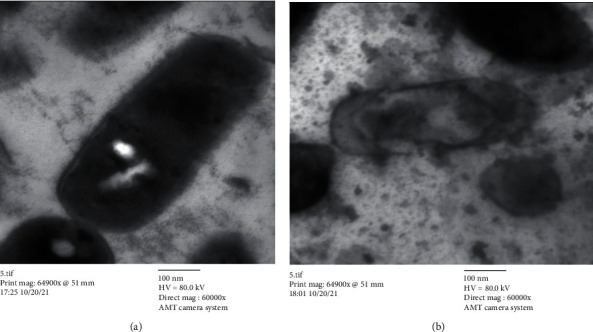
Transmission electron micrograph: (a) untreated *K. pneumoniae* cells (b); *K. pneumoniae* cells treated with *H. perforatum* total methanol extract.

In the current study, there were many ultrastructural alterations in *K. pneumoniae* cells as affected by *H. perforatum* total methanol extract. In the control sample, TEM micrographs (Figure 1(a)) revealed the healthy, thin, and typical ellipse shape of individual *K. pneumoniae* cells, which were surrounded by the inner and outer layers without any cellular injury. The cell membrane of *H. perforatum*-treated *K. pneumoniae* cells was detached from cells, with misshapen cells with loss of structure. This study showed for the first time the ultrastructure alterations involved in the anti-*K. pneumoniae* action by *H. perforatum* total extract (Figure 1(b)).

### 3.4. Antioxidant Activity

**Figure 2:**
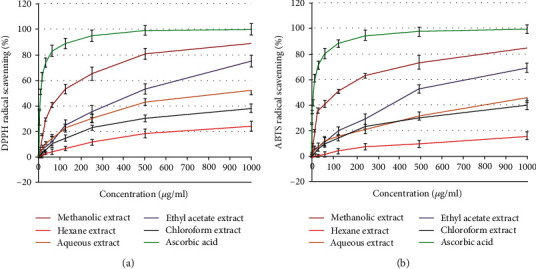
The dose-response curve compares the *in vitro* antioxidant activities of different extracts from *H. perforatum* expressed as (a) DPPH or (b) ABTS radical-scavenging activity percentages at various concentrations (*μ*g/ml) ± SD of three replicates and compared with ascorbic acid as reference standard.

Using the 2,2′-azino-bis(3-ethylbenzothiazoline-6-sulfonic acid) (ABTS) and 2,2-diphenyl-1-picrylhydrazyl (DPPH) tests, the antioxidant activity was assessed as a free radical-scavenging capacity. It has been widely utilized to measure the free radical-scavenging abilities of antioxidants. The DPPH free radical is a stable free radical. The outcomes demonstrated that the studied plant extracts' DPPH or ABTS radical-scavenging abilities were exerted in a dose-dependent manner (Figure 2). Additionally, *H. perforatum*'s methanol extract demonstrated half-maximal inhibitory concentrations (IC_50_) of 98.73 ± 3.59 and 119.86 ± 4.12 *μ*g/ml, respectively. The ethyl acetate extract had exceptional antioxidant potential as indicated by both DPPH and ABTS assays, with IC_50_ values of 467.18 and 489.32 *μ*g/ml, respectively.

### 3.5. Cytotoxic Activity

**Figure 3:**
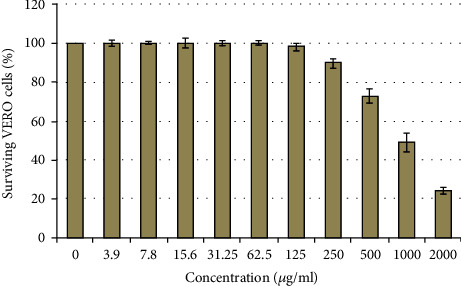
The *in vitro* cytotoxic effects of the bioactive methanol extract from *H. perforatum*. The cytotoxic effects were tested against African green monkey kidney (VERO) cell line using MTT viability assay at different concentrations. Data are expressed as surviving cell percentages at various concentrations (*μ*g/ml) ± SD of three replicates.

It was demonstrated that the methanol extract from *H. perforatum* has negligible cytotoxic effects even at high doses (500 *μ*g/ml or less). Under these screening circumstances, the calculated CC_50_ value was 976.75 *μ*g/ml as depicted in Figure 3.

### 3.6. LC-MS Outcome

**Figure 4:**
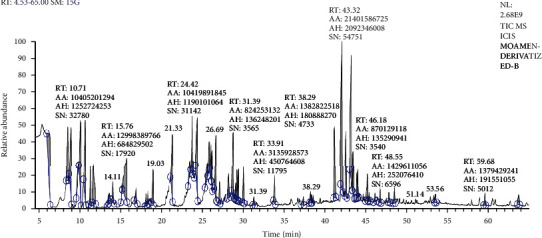
Chromatographic separation of the methanol extract obtained from *H. perforatum* using LC-MS total ion chromatogram showing the separated peaks.

**Figure 5:**
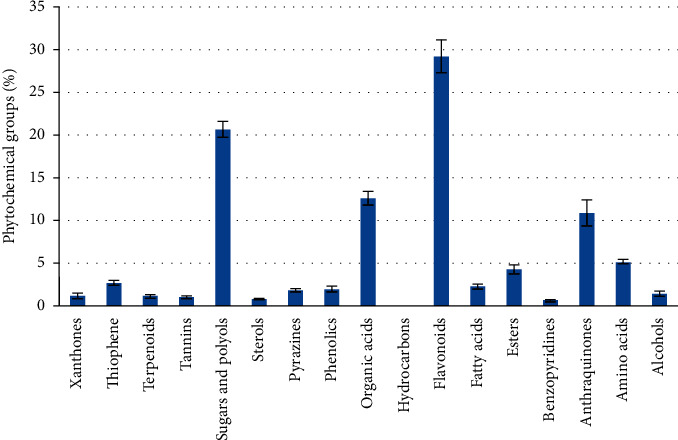
The separated phytochemical groups detected by *H. perforatum* methanol extract under experimental chromatographic conditions.

The relative percentages of the separated chemicals were calculated after the *H. perforatum* methanol extract was chromatographically separated using LC-MS (Table 3, Figure 4). This led to the separation of distinctive fragment ions that were recognized by the system software. According to Table 3, quercetin-3-*β*-D-galactopyranoside came in second with 8.45% of the total LC-MS chromatogram contents, followed by hypericin as an anthraquinone derivative with 10.89%. As indicated in Figure 5, the most prevalent chemical class in the *H. perforatum* extract was flavonoids, followed by sugars and polyols, organic acids, anthraquinones, amino acids, esters, thiophene, fatty acids, and phenolics. Additionally, the isolated chemicals were divided into a total of seventeen phytochemical categories. Furthermore, the lipids were exerted by fatty acids and sterols and represented 3.17% of the total contents. On the other hand, deoxyfructosazine as pyrazines (1.83%), alcohols (1.48%), and xanthones (1.26); pentacyclic triterpenoids (olean-12-en-3-ol derivatives) (1.17%); procyanidins as tannins (1.08%); and 1,3,4-trihydro-2-thioxomethyl-2h-isoquinoline as benzopyridines (0.68) and hydrocarbons (0.03%) were also presented as minor contents.

## 4. Discussion

Novel antimicrobial compounds should be developed to combat the microorganisms that lead to various infections and diseases [27]. Traditional medicine employs *H. perforatum* extract as a treatment for diabetes mellitus, intestinal worms, and snake bites [6]. However, there is still little knowledge about its antibacterial efficacy, particularly when it comes to germs that are resistant to many drugs. As a result, the objective of this study is to assess the antibacterial activity of this plant's aerial portions. The agar well diffusion method was used to investigate the antibacterial activity of the *H. perforatum* whole extract and its four solvent fractions against seven bacterial isolates (2 Gram-positive and 5 Gram-negative bacteria).

In the present study, treatment of bacteria by methanol extract of *H. perforatum* revealed cell shrinkage and distortion where immediate antibacterial activity on the cells was observed to target the elements of the cell membrane. Along with the thickening effect, the cells' size had greatly grown, which caused the cells to elongate. The contraction of the cells caused a considerable reduction in cell size leading to lysing cells. The ultrastructure alternations resemble those seen in prior research using hexane extract of *Halimeda discoidea* against *K. pneumoniae* cells. For example, some cell walls appeared to be broken and caused cytoplasmic leakage, and some cells suffered severe damage, were distorted, and collapsed, which led to a significant decrease in the size of the cell [28].

According to Rajeshwari et al. [29], the cefotaxime antibiotic may have induced the disarray of the cell wall's cross-linking peptidoglycan units and fragmented the cell wall of the tested *K. pneumoniae* by actively attaching to the inactivated penicillin-binding proteins. Additionally, other research groups [30, 31] historically reported that antibiotics like penicillin work by firmly attaching to the enzymes and proteins involved in the elongation and division of Gram-negative cells. Therefore, it was believed that the presence of elongated cells in this investigation would imitate the actions of those two medicines. Although the damaged cells were unable to divide vertically and failed to form septa, they were still able to expand laterally [28].

The methanol extract of *H. perforatum* demonstrated more free radical-scavenging activity than the other extracts. These findings suggest that the *H. perforatum* plant may be an inhibitor of free radicals. Ascorbic acid's capacity to scavenge radicals was compared to the antioxidant capacity of the *H. perforatum* plant in order to scavenge the ABTS radical cation. Comparatively, *H. perforatum* displayed comparatively poor antioxidant activity. Previous investigations have shown that the existence of a functional component supported the antioxidant property of the *H. perforatum* plant [32, 33].

Alcoholic extracts of *H. perforatum* have been shown to have significant antioxidant and radical-scavenging properties, with the flavonoid-rich fraction being one of the key contributors [34]. Benedi et al. [35] investigated the suppression of lipid peroxidation, the hydroxyl radical-scavenging activity, and the interaction with 1,1-diphenyl-2-picrylhydrazyl stable free radical (DPPH) in a standardized extract of *H. perforatum*. According to the present findings, Hypericum extracts exhibit significant antioxidant activity both *in vitro* and in a cell system by preventing the production of free radicals and lipid peroxidation. According to Zou et al. [36] and numerous commercially available formulations, the flavonoid fraction of *H. perforatum* has antioxidative and radical-scavenging properties.

The antioxidative capabilities guard human neuroblastoma cells against apoptosis that is generated [37]. Additionally, Franchi et al. [38] reported that different *H. perforatum* extracts have strong scavenging action, with IC_50_ values ranging from 2.32 to 9.77 *μ*g/ml for the most polar to the most hydrophilic extracts. Surprisingly, *H. perforatum* and hyperforin's methods of action include their capacity to reduce ROS generation and correct pH imbalance in tumor cells [13].

The current study's findings showed that *H. perforatum* had naphthodianthrones, notably hypericin and pseudohypericin, hyperforin, proanthocyanins, flavonoids, biflavonoids, xanthones, phenylpropenes, phenolic acids, and volatile components in accordance with other research groups [39–41]. Gibbons et al. [42] also tested extracts from 34 species and varieties of the genus Hypericum for activity against MRSA where 33 Hypericum extracts showed considerable activity, and five of those extracts had minimum inhibitory doses of 64 *μ*g/ml. Additionally, the low-density lipoprotein (LDL) oxidation systems used hypericin, pseudohypericin, and hyperforin at levels as low as 2.5 *μ*mol/l which are powerful antioxidants [43].

## 5. Conclusions

In this work, *H. perforatum*'s crude extracts and its fractions showed good antibacterial activity with low MICs against drug-resistant bacterial strains especially methanol extract which had anti-*K. pneumonia* action with inhibition zone of 27.9 ± 1.3 and MIC value of 3.9 *μ*g/ml. These results support the use of these extracts as herbal remedies for the management of infections that are resistant to antibiotics. Therefore, before using it in medicine, it is advised to do in-depth investigations including *in vivo* confirmation of antibacterial activity, toxicity, and pharmacokinetics. Additionally, it is possible to perform the separation of active molecules, which will be highly beneficial in modern drug design and may concentrate on pinpointing the precise cellular target(s) and molecular bases of the results seen in this study.

## Figures and Tables

**Table 1:** Resistance profile of multi-drug-resistant isolates.

Microorganism	Resistance pattern of antibiotic agent (R)	Sensitivity pattern of antibiotic agent (S)	Ratio of the no. of resistant antibiotics/total no. of antibiotics
*Methicillin-resistant Staphylococcus aureus*	FOX, PG, OX, IMP, GN, CIP, E, CC, TET, & FA	MOX, LZD, TEC, VA, TET, TGC, RIF, SXT, & FOS	10/19
*Enterococcus faecalis*	PG, AMP, CXM, CXM/AXETIL, GN HL, STREP HL, LEV, MOX, E, CC, Q-D, TET, & SXT	AMP/S, IMP, LZD, TEC, VAN, TGC, & NI	13/20
*Escherichia coli*	TEM, AMP, AMC, PIP, TZP, CF, CXM, CXM-axetil, CTX, CAZ, CRO, FEP, ETP, MEM, CIP, NI, & SXT	AK, GN, TGC, FOS, & CT	18/23
*Klebsiella pneumoniae*	TEM, AMP, AMC, PIP, TZP, CF, CXM, CXM-axetil, CTX, CAZ, CRO, FEP, ERTP, AK, GN, CIP, IMP, MEM, & NI	TGC, FOS, CT, & SXT	19/23
*Pseudomonas aeruginosa*	TIC, TCC, PIP, TZP, CAZ, FEP, IMP, MEM, AK, GN, TM, CIP, PEF, MNO, CT, SXT	—	16/16
*Acinetobacter baumannii*	TEM, AMP, AMC, PIP, TZP, CF, CXM, CXM-axetil, CTX, CAZ, FEP, IMP, MEM, GN, CIP, FOS, NI, CRO, ERTP, & SXT	AK, TGC, & CT	20/23
*Proteus mirabilis*	AMP, PIP, CF, CXM, CXM-axetil, CTX, CAZ, CRO, FEP, CIP, FOS, NI, SXT, TGC, & CT	TEM, AMC, TZP, ERT, IMP, MEM, AK, & GN	15/23
*Stenotrophomonas maltophilia*	TEM, AMP, AMC, PIP, TZP, CF, CXM, CXM-axetil, CTX, CAZ, CRO, FEP, ETP, IMP, MEM, AK, GN, CIP, TGC, FOS, NI, & SXT	CL	22/23

Used antibiotics: A/S = ampicillin/sulbactam; AK = amikacin; AMC = amoxicillin/clavulanic acid; AMP = ampicillin; CAZ = ceftazidime; CF = cephalothin; CIP = ciprofloxacin; CLN = clindamycin; CRO = ceftriaxone; CT = colistin; CTX = cefotaxime; CXM = cefuroxime; CXM-axetil = cefuroxime axetil; E = erythromycin; ETP = ertapenem; FD = fusidic acid; FEP = cefepime; FOS = fosfomycin; FOX = cefoxitin; GN = gentamicin; GN HL = gentamicin high level; ICR = inducible clindamycin resistance; IMP = imipenem; LEV = levofloxacin; LZD = linezolid; MEM = meropenem; MNO = minocycline; MOX = moxifloxacin; NI = nitrofurantoin; OX = oxacillin; P = benzylpenicillin; PEF = pefloxacin; PIP = piperacillin; Q-D = quinupristin/dalfopristin; RIF = rifampicin; STREP HL = streptomycin high level; SXT = trimethoprim/sulfamethoxazole; TCC = ticarcillin/clavulanic acid; TEC = teicoplanin; TEM = temocillin; TET = tetracycline; TGC = tigecycline; TIC = ticarcillin; TM = tobramycin; TZP = piperacillin/tazobactam; VAN = vancomycin.

**Table 2:** Susceptibility of MDR isolates to different *Hypericum perforatum* extracts and MIC values for *Hypericum perforatum* extracts against MDR strains tested at 10 mg/ml using agar well diffusion assay.

Microorganisms	Extracts					
	Methanol extract	n-Hexane	Ethyl acetate	Chloroform	Aqueous	MIC of methanol extract (*μ*g/ml)
	Mean of inhibition zones (mm)					
MRSA	23.0 ± 0.82	NIZ	22.3 ± 0.74	16.2 ± 0.17	19.3 ± 0.57	15.63
*E. faecalis*	25.3 ± 0.44	NIZ	19.8 ± 0.92	20.9 ± 0.37	18.4 ± 0.64	3.9
*E. coli*	19.31 ± 1.1	12.4 ± 0.85	18.9 ± 1.39	NIZ	15.2 ± 2.1	62.5
*K. pneumoniae*	27.9 ± 1.3	NIZ	24.45 ± 1.23	18.6 ± 1.24	14.8 ± 0.58	3.9
*P. aeruginosa*	20.42 ± 0.8	NIZ	16.38 ± 1.14	16.35 ± 1.61	NIZ	31.25
*A. baumannii*	NIZ	NIZ	NIZ	NIZ	NIZ	—
*P. mirabilis*	17.93 ± 0.25	15.3 ± 0.37	16.3 ± 0.63	NIZ	13.4 ± 0.28	125
*S. maltophilia*	18.3 ± 0.37	NIZ	15.8 ± 0.51	18.6 ± 0.52	13.4 ± 0.37	62.5

NIZ: no inhibition zones; MIC = minimum inhibitory concentration; (–) = no activity of the extracts tested.

**Table 3:** The constituent's identification of the separated metabolites obtained from methanol extract of *H. perforatum* using LC/MS chromatographic separation technique.

Peak no.	Retention time (min)	Constituent identification	Relative content (%)	Molecular formula	*m*/*z*
1	6.40	L-Homocitrulline	5.20	C_7_H_15_N_3_O_3_	188.9
2	8.60	Thiophene,2-[2-(3-methylphenyl)-ethenyl]-	2.71	C_13_H_12_S	200.1
3	8.99	2-Methyl-4-keto-pentan-2-ol	3.63	C_11_H_26_O	188.2
4	10.14	Butanoic acid	3.50	C_10_H_24_O_3_	88.41
5	10.71	Fumaric acid	3.86	C_10_H_20_O_4_	116.07
6	11.35	1,4-Butanediol	1.48	C_10_H_26_O_2_	90.12
7	11.73	Malic acid	3.32	C_13_H_30_O_5_	134.08
8	14.11	1,3,6,7-Tetrahydroxyxanthone	1.26	C_13_H_8_O_6_	259.2
9	15.76	Kaempferol	4.95	C_15_H_10_O_6_	287.05
10	16.95	Luteolin	1.34	C_15_H_10_O_6_	285.1
11	19.03	Erythritol	1.76	C_16_H_42_O_4_	122.12
12	21.34	Quercetin	2.71	C_15_H_10_O_7_	303.05
13	23.81	D-Fructofuranose	2.76	C_21_H_52_O_6_	179.16
14	24.42	Quercetin-3-O-*α*-L-arabinofuranoside	4.96	C_20_H_18_O_11_	434.3
15	25.87	D-Glucitol	2.33	C_24_H_62_O_6_	434.39
16	26.09	Quininic acid	1.97	C_22_H_52_O_6_	533.07
17	26.22	Procyanidin B2	1.08	C_30_H_26_O_12_	577.02
18	26.69	Myo-Inositol	2.42	C_24_H_60_O_6_	180.16
19	28.27	D-Allofuranose	1.04	C_21_H_52_O_6_	260.07
20	28.79	D-Gluconic acid	2.97	C_24_H_60_O_7_	629.24
21	29.30	D-Glucopyranose	1.23	C_21_H_52_O_6_	180.15
22	30.09	Gulonic acid, ç-lactone	1.58	C_18_H_42_O_6_	466.86
23	31.39	Nonadecanoate (ester)	0.61	C_18_H_42_O_6_	312.51
24	33.91	Palmitic acid	1.19	C_19_H_40_O_2_	255.34
25	37.40	1,3,4-Trihydro-2-thioxomethyl-2 h-isoquinoline	0.68	C_10_H_11_N	177.08
26	38.29	Stearic acid	0.73	C_21_H_44_O_2_	356.66
27	41.27	*α*-D-Glucopyranoside	3.67	C_36_H_86_O_11_	341.11
28	42.21	Hypericin	10.89	C_30_H_16_O_8_	503.08
29	42.64	3-O-Coumaroyl-D-quinic acid	5.24	C_31_H_58_O_8_	699.21
30	43.32	Quercetin-3-*β*-D-galactopyranoside	8.45	C_28_H_24_O_16_	487.08
31	43.53	Deoxyfructosazine	1.83	C_33_H_76_N_2_O_7_	304.30
32	44.05	Hyperforin	1.65	C_35_H_52_O_4_	536.8
33	45.24	Rutin	1.59	C_27_H_30_O_16_	611.16
34	46.18	Behenic acid	0.38	C_25_H_52_O_2_	340.62
35	46.81	Lactose	0.52	C_36_H_86_O_11_	918.02
36	48.55	D-(+)-Cellobiose	0.34	C_37_H_89_NO_11_	342.31
37	51.14	cis-5-O-Feruloylquinic acid	0.41	C_32_H_60_O_9_	729.2
38	53.56	Chlorogenic acid	1.62	C_34_H_66_O_9_	354.31
39	59.68	*α*-Amyrin = olean-12-en-3-ol	0.53	C_33_H_58_O	426.72
40	60.75	Olean-12-en-3-ol, acetate	0.64	C_32_H_52_O_2_	468.8
41	63.78	Beta-sitosterol	0.87	C_29_H_50_O	576.83

## Data Availability

On reasonable request, the accompanying authors will provide all data that support the study's conclusions.

## Abbreviations

MDR: Multi-drug-resistant
MRSA: Methicillin-resistant *Staphylococcus aureus*
MIC: Minimal inhibitory concentration
LC-ESI-MS/MS: Liquid chromatography electrospray ionization tandem mass spectrometric analysis
DPPH: 2,2-Diphenyl-1-picrylhydrazyl
ABTS: 2,2′-Azino-bis(3-ethylbenzothiazoline-6-sulfonic acid) (a chemical compound used to observe the reaction kinetics of specific enzymes)
TEM: Transmission electron microscopy
ROS: Reactive oxygen species.
